# 8-Methoxyflindersine-Induced Apoptosis and Cell Cycle Disorder Involving MAPK Signaling Activation in Human Colorectal Cancer Cells

**DOI:** 10.3390/ijms24098039

**Published:** 2023-04-28

**Authors:** Dianbao Zhang, Yunmei Fu, Ying Liu, Yifan Wu, Jiayu Chen, Luting Zhang, Rui Wang, Zaixing Chen, Tao Liu

**Affiliations:** 1 Department of Stem Cells and Regenerative Medicine, Key Laboratory of Cell Biology, National Health Commission of China, and Key Laboratory of Medical Cell Biology, Ministry of Education of China, China Medical University, Shenyang 110122, China; zhangdianbao@gmail.com (D.Z.); 2021120051@cmu.edu.cn (Y.L.); 2022120047@cmu.edu.cn (Y.W.); chenjiayu@cmu.edu.cn (J.C.); rwang@cmu.edu.cn (R.W.); 2 Department of Natural Products Chemistry, School of Pharmacy, China Medical University, Shenyang 110122, China; fym961104@163.com (Y.F.); luting_zhang@163.com (L.Z.); 3 Central Laboratory, School of Pharmacy, China Medical University, Shenyang 110122, China

**Keywords:** 8-methoxyflindersine, colorectal cancer, apoptosis, cell cycle, MAPK signaling

## Abstract

Colorectal cancer (CRC) is one of the most common malignant tumors with a high lethal rate globally, and novel strategies for its prevention and therapy are urgently needed. In our previous work, 8-methoxyflindersine (8-MF), a quinoline alkaloid, was isolated from the Dictamni cortex, and its bioactivities were largely unknown. In this study, we found that 8-MF significantly inhibited cell viability in the CRC cell lines LoVo and RKO. The 8-MF-induced CRC cell apoptosis, as well as cell cycle disorder, were further verified by cyclins dysregulation in mRNA and protein levels. Further, the activation of MAPK family members p38 and ERK1/2 was observed after 8-MF treatment. Moreover, the protein–protein interaction of 8-MF with cyclins and MAPKs was demonstrated using the STRING database. The 8-MF could bind to p38 and ERK1/2 proteins in molecular docking. Taken together, we found that 8-MF induced apoptosis and cell cycle disorder involving MAPK signaling activation in CRC cells, indicating 8-MF as a novel lead compound candidate for the development of anti-tumor drugs for CRC.

## 1. Introduction

Colorectal cancer (CRC) is the third most commonly diagnosed cancer and the second leading cause of cancer death worldwide [1]. Serrated polyps are considered to be precursors to CRC [2]. The common treatments in the early stage are surgical resection and systemic chemotherapy [3]. Chemical adjuvant therapy of multiple-agent combinations has also been proven to be effective, and the standard treatment options are fluorouracil or capecitabine with or without oxaliplatin [4]. However, drug resistance in CRC remains a great clinical challenge [5]. Considering this, there comes an urgent need to explore more novel strategies for the prevention and therapy of CRC.

The Dictamni cortex, also known as Bai-Xian-Pi in Chinese, is the dried root bark of *Dictamnus dasycarpus*. The Dictamni cortex, according to traditional Chinese medical theories, is able to dispel wind and relieves itching, which are quite useful in allergic reactions and skin pruritus. [6]. Several studies have reported that the Dictamni cortex has anti-hepatotoxicity [7], anti-inflammatory [8], and anti-tumor activities [9]. Furthermore, it has been suggested that natural products derived from the Dictamni cortex could be exploited as sources of anticancer drugs [10]. However, its biological activities remain largely unexplored and no substantial evidence demonstrates that natural products derived from the Dictamni cortex [6] can be used for CRC therapy.

The 8-methoxyflindersine (8-MF) was a quinoline alkaloid isolated from several plants, such as *Conchocarpus fontanesianus* [11] and *Dictyoloma vandellianum* [12], and we have obtained 8-MF from the Dictamni cortex recently [6]. The research on 8-MF was primarily focused on separation [13], synthesis [14], and chemotaxonomic significance [15], in which its anticancer activity is unclear.

In this study, the cytotoxicity of 8-MF on various human cancer cells was evaluated, and cell apoptosis and cell cycle disorder were found to be responsible for its inhibitory effects on CRC cells. The involvement of MAPK signaling was further investigated.

## 2. Results

### 2.1. 8-MF Reduced CRC Cells Viability

**Figure 1:**
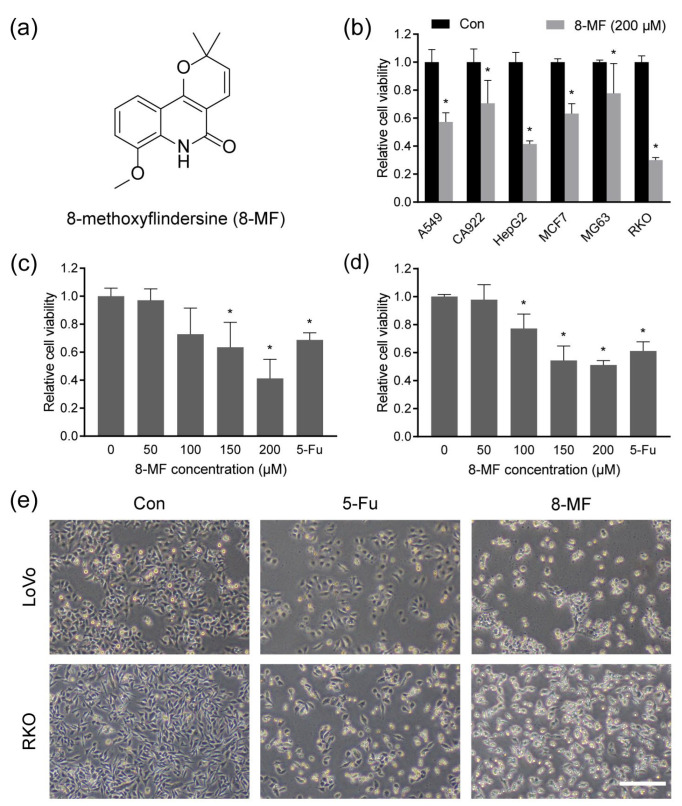
8-MF reduced CRC cell viability. (**a**) The chemical structure of 8-methoxyflindersine (8-MF); (**b**) Several human cancer cells were treated with 8-MF at 200 µM for 48 h, and the cell viability was measured using the CCK-8 assay; (**c**) The cytotoxicity of 8-MF on LoVo cells at different concentrations (0, 50, 100, 150, and 200 µM) was analyzed using the CCK-8 assay, with 200 µM 5-Fu serving as the positive control; (**d**) The cytotoxicity of 8-MF on RKO cells at different concentrations (0, 50, 100, 150, and 200 µM) was analyzed using the CCK-8 assay, with 200 µM 5-Fu serving as the positive control; (**e**) The representative images of LOVO and RKO cells treated with 5-Fu and 8-MF at 200 μM for 24 h, bar indicates 200 μm. * *p* < 0.05, compared with 0 μM groups, *n* = 5.

The chemical structure of 8-MF is shown in Figure 1a. To assess the anticancer potential of 8-MF, the CCK-8 assay was carried out on six cancer cell lines, including human lung cancer cell A549, oral squamous carcinoma cell CA922, hepatocellular carcinoma cell HepG2, breast cancer cell MCF7, osteosarcoma cell MG63, and CRC cell RKO. The cells were treated with 8-MF at 200 μM for 48 h and the viabilities of these cells were reduced significantly. The inhibitory effect of 8-MF on the CRC cell line RKO was stronger than that on others (Figure 1b). Subsequently, the cytotoxicity of 8-MF on RKO and another CRC cell LoVo at different concentrations (0, 50, 100, 150, and 200 μM) was analyzed, with 200 µM 5-Fu serving as a positive control. As shown in Figure 1c,d, the cell viabilities of LoVo and RKO were inhibited by 8-MF in a concentration-dependent manner, and the values of IC_50_ were 176.8 and 181.6 μM, respectively. The inhibitory activity of 8-MF on CRC cells was comparable to that of 5-Fu. In addition, LoVo and RKO cells presented spherical and shrunken morphology and decreased cell numbers after the treatment with 8-MF at 200 μM for 24 h (Figure 1e). These results indicated 8-MF as a novel lead compound candidate for the treatment of CRC.

### 2.2. 8-MF–Induced CRC Cells Apoptosis

**Figure 2:**
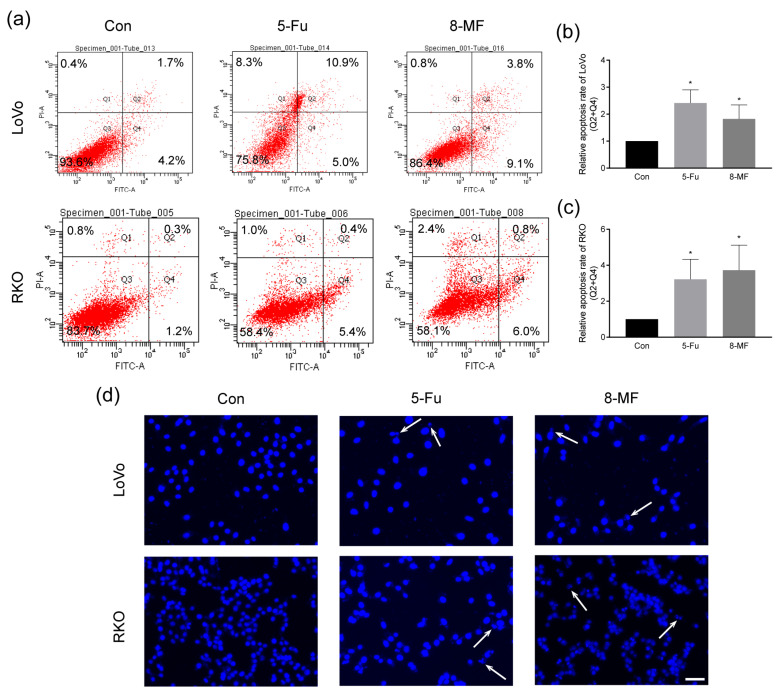
8-MF–induced CRC cell apoptosis. RKO and LoVo cells were treated with 200 μM 8-MF or 5-Fu for 24 h, respectively. (**a**–**c**) The CRC cell apoptosis upon 200 µM 8-MF or 5-Fu treatment for 24 h was analyzed using Annexin V-FITC/PI staining followed by flow cytometry; (**d**) The nucleus morphology was visualized by DAPI staining. Arrows indicate pyknosis and karyorrhexis. Bar indicates 100 μm. * *p* < 0.05, compared with Con groups, *n* = 3.

The CRC cell apoptosis upon 8-MF treatment was analyzed using Annexin V-FITC/PI staining followed by flow cytometry. The proportions of early apoptosis cells were increased after exposure to 200 μM 8-MF for 24 h in LoVo and RKO cells (Figure 2a–c). Furthermore, the nucleus morphology was visualized by DAPI staining, and pyknosis and karyorrhexis were observed in 8-MF treated cells (Figure 2d). These data suggested that 8-MF could induce CRC cell apoptosis.

### 2.3. 8-MF–Induced CRC Cell Cycle Disorder

**Figure 3:**
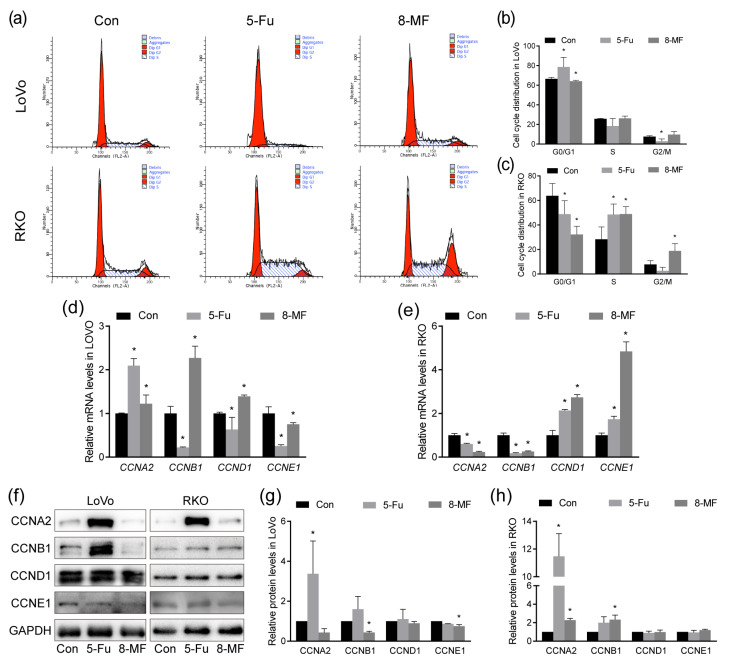
8-MF–induced cell cycle disorder and dysregulation of cyclins expression in CRC cells. RKO and LoVo cells were treated with 200 μM 8-MF or 5-Fu for 24 h, respectively. (**a**–**c**) The cell cycle distribution was analyzed by PI staining followed by flow cytometry; (**d**) The mRNA levels of cyclins in LoVo cells were analyzed by real-time PCR; (**e**) The mRNA levels of cyclins in RKO cells were analyzed by real-time PCR; (**f**–**h**) The protein expression of cyclins was analyzed by western blotting. The images were analyzed using ImageJ 1.52a software. * *p* < 0.05, compared with control groups, *n* = 3.

To explore the cell cycle distribution of CRC cells upon 8-MF treatment, PI staining followed by flow cytometry was carried out. As shown in Figure 3a–c, after treatment with 8-MF for 24 h, LoVo cells in the G0/G1 phase were decreased while RKO cells were accumulated in the S phase and G2/M phase. The results of real-time PCR showed that 8-MF enhanced mRNA levels of *CCNB* and *CCND* in LoVo cells (Figure 3d) and reduced *CCNA* and *CCNB* while enhancing *CCND* and *CCNE* in RKO cells (Figure 3e). However, the protein expression in western blotting results presented a different pattern. After 8-MF treatment, the expression of CCNA and CCNB was reduced in LoVo cells but enhanced in RKO cells (Figure 3f–h). Collectively, 8-MF induced cell cycle disorder and cyclins expression dysregulation in CRC cells.

### 2.4. MAPK Signaling Was Involved in 8-MF-Induced Inhibition of CRC Cells

**Figure 4:**
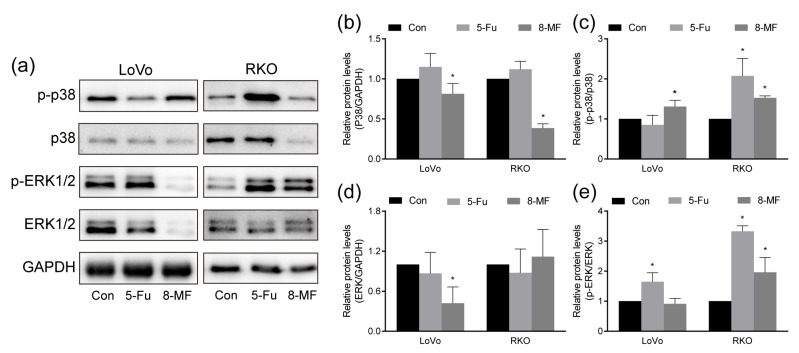
The involvement of MAPK signaling in 8-MF–induced CRC inhibition. LoVo and RKO cells were treated with 8-MF and 5-Fu at 200 μM for 24 h, respectively. (**a**) The total and phosphorylated protein levels of MAPK signaling pathways were detected using western blotting; (**b**–**e**) The bands of western blotting results were analyzed using ImageJ 1.52a. * *p* < 0.05, compared with control groups, *n* = 3.

The ubiquitous MAPK signaling regulates almost all cell behaviors, including the tumorigenesis and development of CRC. Here, the expression and phosphorylation of MAPK family members p38 and ERK1/2 (p44/p42) were detected using western blotting. The phosphorylated p38 was elevated by 8-MF treatment in LoVo and RKO cells; meanwhile, the expression of total p38 was reduced (Figure 4a–c). In LoVo cells, the total ERK1/2 expression was inhibited, and its activation by phosphorylation was not affected by 8-MF treatment, while in RKO cells, the phosphor-ERK1/2 was enhanced and the total ERK1/2 was not changed upon 8-MF incubation (Figure 4a,d,e). These findings established the role of MAPK signaling in the 8-MF-induced inhibition of CRC cells.

### 2.5. Interactions between 8-MF and Proteins

**Figure 5:**
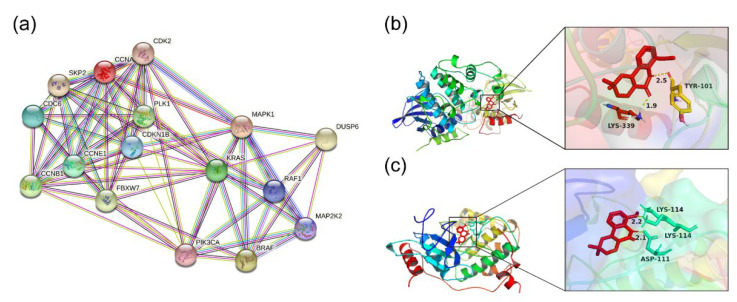
Computer models predicted the relationship between cyclins, MAPK signaling, and 8-MF. (**a**) STRING bioinformatics analysis for PPI between cyclins and MAPK signaling; (**b**) The molecular docking model of 8-MF with p38 protein; (**c**) The molecular docking model of 8-MF with ERK protein.

To further explore the mechanism by which 8-MF inhibits CRC cells, STRING and molecular docking were used. STRING predicted the functional associations between cyclins and MAPK signaling. The result revealed protein–protein interactions (PPI) among CCNA2, CCNB1, CCNE1, and ERK (MAPK1), as well as KRAS as a mediator hub (Figure 5a). To further explore the potential mechanism involved, molecular docking was used to predict the interaction between 8-MF and related proteins based on spatial structure and energy scoring. The molecular docking models were created using AutoDock 4.2.6. As is shown in Figure 5b,c and Table 1, 8-MF contact tyrosine (TYR-101) and lysine (LYS-339) of p38 protein (−7.13 kcal/mol), and contact aspartic acid (ASP-111) and lysine (LYS-114) of ERK protein (−7.35 kcal/mol). These findings suggested that 8-MF could interact with p38 and ERK to modulate their activation, promoting cell cycle disorder to inhibit CRC cells.

## 3. Discussion

CRC is one of the most common forms of digestive cancer in the globe [16]. The main treatment is surgical resection followed by chemotherapy [17]. Increasing evidence suggests that natural products, including quinoline alkaloids [18], have attracted much attention due to their therapeutic potential for cancer treatment [19]. The 8-MF, a quinoline alkaloid, has potential as an effective anti-tumor treatment and was isolated from the Dictamni cortex in our previous study [6]. Additionally, the results of the in vitro screening presented that 8-MF had strong inhibitory effects on CRC cells. Consequently, the effects of 8-MF on CRC cells and their underlying mechanisms were explored in this study.

The results we obtained using CCK-8 assay presented that CRC cell proliferation was inhibited upon 8-MF treatment, suggesting that 8-MF had a potent anti-proliferative effect on CRC cells. Then cell cycle disorder and apoptosis were found to contribute to 8-MF–induced inhibitory effects involving the MAPK signaling pathway. Cell cycle regulation plays a pivotal role in cancer treatment by traditional Chinese medicine, for example, esculetin-induced cell cycle arrest in LoVo cells [20]. In this study, 8-MF induced S and G2/M phase arrest in CRC cells and was further verified by the dysregulation of cyclins. Interestingly, in LoVo and RKO cells, the cell cycle was not arrested at the same pattern. The KRAS mutations in LoVo cells and v-raf murine sarcoma viral oncogene homolog B1 (BRAF) mutations in RKO cells [21] might contribute. The GTPase RAS protein family member KRAS and its downstream effector serine–threonine kinase BRAF play critical roles in the regulation of the cell cycle and MAPK axis. Their mutations were discovered as predictors of colorectal carcinogenesis and resistance to EGFR-targeted therapy [22]. These findings indicated that 8-MF inhibited CRC cells regardless of whether KRAS and BRAF were mutant or not, although their mutations perturb the inhibitory pathways of 8-MF. However, the mechanisms involved remain to be explored in depth.

MAPK signaling pathways regulate cell proliferation, differentiation, migration, senescence, and apoptosis [23]. Here, we investigated the involvement of MAPK signaling pathways in 8-MF-induced antiproliferation effects in LoVo and RKO cells. According to the findings, 8-MF activated p38 and ERK1/2 while reducing their expression in LoVo and RKO cells. Moreover, computational molecular docking models revealed that 8-MF bound well to proteins p38 and ERK. These results suggested that MAPK signaling was the primary pathway mediating 8-MF-induced inhibition of CRC cells.

**Figure 6:**
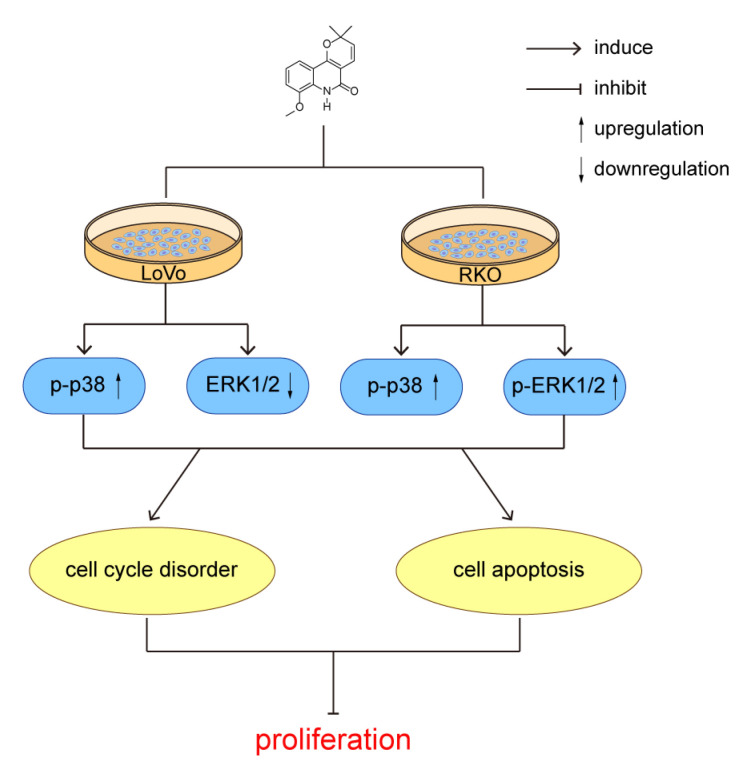
A schematic presentation about the molecular mechanism that 8-MF induced CRC cell apoptosis and cell cycle disorder, involving MAPK signaling activation.

In summary, it is significant that 8-MF induced CRC cell apoptosis and cell cycle disorder involving MAPK signaling activation (Figure 6). As far as we know, it is the first report about the roles of 8-MF in the field of pharmacology. With further studies, 8-MF could be considered a potential novel natural candidate for the treatment of CRC.

## 4. Materials and Methods

### 4.1. Chemicals and Reagents

The isolation of 8-MF (CAS number 35989-00-5, purity ≥ 99.0%) from the Dictamni cortex was described in our previous study [6]. The positive control 5-fluorouracil (5-Fu, purity ≥ 99.0%, ST1060), 4% paraformaldehyde fix solution (P0099), cell cycle and apoptosis analysis kit (C1052), DCFH-DA probe (S0033S), RIPA buffer (P0013B), protease and phosphatase inhibitor cocktail (P1045) were purchased from Beyotime (Shanghai, China). The 8-MF and 5-Fu were dissolved in DMSO separately and stored at −20 °C. Cell counting Kit-8 (CCK-8, MA0218) and penicillin–streptomycin solution (MA0110) were purchased from Meilunbio (Dalian, China). RPMI 1640 medium was purchased from KeyGEN (KGM31800N-500, Jiangsu, China). FBS was obtained from Biological Industries (04-001-1A, Beit Haemek, Israel). DMEM/high glucose (C11995500BT), RevertAid First Strand cDNA Synthesis Kit (K1622), PowerUp SYBR Green Master Mix (A25778), DAPI (D1360), and Annexin V-FITC Apop Kit (BMS500FI-300) were purchased from Thermo Fisher (Shanghai, China). RNAiso Plus (9108) and BCA Protein Assay Kit (T9300A) were obtained from TaKaRa (Dalian, China). The ECL Prime Western Blotting Detection Reagent was obtained from GE Healthcare (RPN2236, IL, USA). The antibodies against GAPDH (60004-1-Ig), CCNA (18202-1-AP), CCNB (28603-1-AP), CCND (60186-1-Ig), CCNE (11554-1-AP), and the secondary antibodies were purchased from Proteintech (Wuhan, China). Antibodies against p38 (8690S), phospho-p38 (4511S), ERK1/2 (p44/42) (4595S), and phospho-ERK1/2 (p44/42) (4370S) were purchased from Cell Signaling Technology (Shanghai, China).

### 4.2. Cell Culture

The human CRC cell lines LoVo and RKO were obtained from Shanghai Institutes for Biological Sciences (Shanghai, China). The LoVo and RKO cells and the non-small cell lung cancer cell A549 were cultured in RPMI 1640 medium supplemented with 10% FBS and 1% penicillin–streptomycin solution. Human oral squamous carcinoma cell CA922, hepatocellular carcinoma cell HepG2, breast cancer cell MCF-7, and osteosarcoma cell MG63 were purchased from Procell (Wuhan, China) and cultured in DMEM/high glucose with 10% FBS and 1% penicillin–streptomycin solution. All cells were incubated at 37 °C with 5% CO_2_. The cell morphology was observed using an inverted phase contrast microscope (CKX41, Olympus, Beijing, China).

### 4.3. Cell Viability Assay

The cells were seeded into 96-well plates at 1 × 10^4^ cells per well and incubated overnight. The cells were then treated with 8-MF and 5-Fu at indicated concentrations for 24 and 48 h. The CCK-8 reagent (10 μL) was added to each well, and the cells were incubated at 37 °C for 1 h. The absorbance at 450 nm was measured using a microplate reader (iMark, Bio-Rad Laboratories, Hercules, CA, USA) with a reference wavelength of 630 nm.

### 4.4. DAPI Staining Assay

The cells were seeded into 24-well plates and treated with 200 μM 8-MF or 5-Fu for 24 h. The cells were then fixed with 4% paraformaldehyde fix solution, washed twice with PBS, and stained with 300 nM DAPI for 3 min in the dark. The cells were rinsed three times with PBS and imaged using an inverted fluorescence microscope (Observer A1, ZEISS, Jena, Germany).

### 4.5. Annexin V-FITC/PI Apoptosis Assay

The cell apoptosis was detected using Annexin V-FITC/PI staining. The cells were seeded into 6-well plates at 1 × 10^5^ cells per well and cultured overnight. The cells were treated with 200 μM 8-MF or 5-Fu for 24 h. Following the manufacturer’s protocol, attached and floating cells were collected by centrifuging and stained with 5 μL Annexin V-FITC and 10 μL PI for 15 min in the dark at room temperature. The cells were analyzed using flow cytometry analysis (Fortessa, BD Biosciences, Franklin Lakes, NJ, USA).

### 4.6. Cell Cycle Analysis

The cell cycle distribution was determined using the Cell Cycle and Apoptosis Analysis Kit. The cells were cultured in 6-well plates and treated with 200 μM 8-MF or 5-Fu for 24 h. According to the manufacturer’s instructions, both attached and floating cells were collected and fixed with 75% ethanol at 4 °C overnight. The cells were incubated with RNase and PI at 37 °C for 30 min. The cells were analyzed using FACSCalibur Flow Cytometry (BD Biosciences).

### 4.7. Real-Time PCR

The cells were cultured in 6-well plates and treated with 200 μM 8-MF or 5-Fu for 24 h. The total RNA was extracted using RNAiso Plus. The reverse transcription and real-time PCR were carried out according to the instructions of the RevertAid First Strand cDNA Synthesis Kit and PowerUp SYBR Green Master Mix. Real-time PCR was performed on the ABI 7500 Real-Time PCR System, and the relative gene expression was analyzed using the 2^−ΔΔCt^ method. GAPDH was used as an internal control. The primers were synthesized by GenScript (Nanjing, China), and the sequences are listed in Table 2.

### 4.8. Western Blotting

The cells were cultured in 6-well plates and treated with 200 μM 8-MF or 5-Fu for 24 h. The protein lysates were prepared using RIPA buffer supplemented with protease and phosphatase inhibitor cocktail and quantified by BCA Protein Assay Kit. The protein samples were separated by SDS-PAGE and transferred onto the PVDF membranes. The membranes were blocked with 5% skim milk and incubated with primary antibodies at 4 °C overnight. After incubation with secondary antibodies for 1 h at room temperature, the protein bands were visualized using the ECL Prime Western Blotting Detection Reagent and imaged under the chemiluminescence detection system (Tanon-5200, Tanon, Shanghai, China). The protein bands were analyzed using ImageJ software (NIH, Bethesda, MD, USA).

### 4.9. Protein–Protein Interaction and Molecular Docking

The protein–protein interaction (PPI) networks between cyclins and MAPK signaling proteins were analyzed using STRING (https://cn.string-db.org/, accessed on 23 April 2022) with the default parameters. To predict the binding of 8-MF to p38 and ERK, molecular docking was performed using Autodock 4.2.6. The protein structure of p38 (protein ID: 4MYG) and ERK (protein ID: 2Y9Q) were downloaded from Protein Data Bank (https://www.rcsb.org/, accessed on 6 May 2022). All of the water atoms were removed, and hydrogen atoms were added. The 3D structure of 8-MF was constructed using Chem3D 17.1. The binding modes and their respective binding energy were obtained after docking. The lowest binding energy conformation was selected and visualized by PyMOL 2.5.4.

### 4.10. Statistical Analysis

The data were presented as the mean ± standard deviation (SD). Statistical evaluation was performed using the Student’s *t*-test for two groups and one-way analysis of variance (ANOVA) followed by Tukey’s post hoc test for multiple comparisons for more than two groups. *p* < 0.05 was considered statistically significant.

## Figures and Tables

**Table 1:** Scores of docking function of 8-MF with p38 and ERK proteins.

Compound	Molecular Formula	Target Name	PDB ID	Minimum Binding Energy (kcal/mol)
8-methoxyflindersine (8-MF)	C_15_H_15_NO_3_	p38	4MYG	−7.13
		ERK	2Y9Q	−7.35

**Table 2:** Primer sequences for real-time PCR.

Gene	Primer Sequence
*GAPDH*	forward:5′-GCACCGTCAAGGCTGAGAAC-3′ reverse:5′-TGGTGAAGACGCCAGTGGA-3′
*CCNA2*	forward:5′-CCTGGACCCAGAAAACCATT-3′ reverse:5′-AACACTCACTGGCTTTTCATCT-3′
*CCNB1*	forward:5′-ACCTGTGTCAGGCTTTCTCT-3′ reverse:5′-TTGGTCTGACTGCTTGCTCTT-3′
*CCND1*	forward:5′-GCTGCGAAGTGGAAACCATC-3′ reverse:5′-CCTCCTTCTGCACACATTTGAA-3′
*CCNE1*	forward:5′-AAGGAGCGGGACACCATGA-3′ reverse:5′-ACGGTCACGTTTGCCTTCC-3′

## Data Availability

The data generated during the current study are available from the corresponding author upon reasonable request.
